# Draft Genome Sequence of *Comamonas thiooxydans* Strain PHE2-6 (NBRC 110656), a Chlorinated-Ethene-Degrading Bacterium

**DOI:** 10.1128/genomeA.00487-16

**Published:** 2016-06-23

**Authors:** Jun Shimodaira, Kenta Yonezuka, Michiro Tabata, Shun Nagase, Daisuke Kasai, Akira Hosoyama, Atsushi Yamazoe, Nobuyuki Fujita, Masao Fukuda

**Affiliations:** a Biological Resource Center, National Institute of Technology and Evaluation, Nishihara, Shibuya-ku, Tokyo, Japan; b Department of Bioengineering, Nagaoka University of Technology, Kamitomioka, Nagaoka, Niigata, Japan

## Abstract

*Comamonas thiooxydans* strain PHE2-6 (NBRC 110656), which was isolated from a trichloroethene-contaminated site in Japan, utilizes phenol as a sole source of carbon and cometabolizes *cis-* and *trans-*dichloroethenes. We report here the draft genome sequence of this strain, containing 5,309,680 bp, with 60.6% G+C content.

## GENOME ANNOUNCEMENT

Chlorinated ethenes, such as trichloroethene (TCE), *cis-*dichloroethene (cDCE), and *trans-*dichloroethene (tDCE), are known as toxic and persistent environmental pollutants in soil and groundwater. A variety of aerobic bacteria, which can degrade chlorinated ethenes, have been isolated, and most of them cometabolize chlorinated ethenes in the presence of methane (1), toluene (2), ammonia (3), or phenol (4). Studies on tDCE degradation by aerobic bacteria are considerably fewer than those on TCE and cDCE. Strain PHE2-6 was isolated from a TCE-contaminated site in Japan and grew on phenol as a sole carbon source. It cometabolized tDCE as well as TCE and cDCE during growth on phenol. To obtain insight into the genomic basis of microbial degradation of phenol and chlorinated ethenes by strain PHE2-6, draft genome sequencing of this strain was performed.

The genomic DNA of strain PHE2-6 was sequenced by using paired-end sequencing with an Illumina MiSeq platform (Illumina, San Diego, CA, USA). The 5,744,311 reads obtained were assembled by Newbler version 2.6. The contigs were analyzed using the NCBI Prokaryotic Genome Annotation Pipeline (http://www.ncbi.nlm.nih.gov/genome/annotation_prok) to annotate protein-coding, rRNA, and tRNA genes. The draft genome of strain PHE2-6 has a total size of 5,306,578 bp, with 61.5% G+C content, and consists of 48 contigs ranging from 641 to 575,292 bp, with an average coverage of 151× and a *N*_50_ length of 224,522 bp. The annotation revealed 4,721 protein-coding sequences and 58 RNA genes containing single copies of 16S and 23S rRNA genes and 56 tRNA genes.

The strain PHE2-6 genome included a single gene cluster involved in phenol and TCE degradation, which contained the orthologs of the multicomponent phenol hydroxylase subunit genes, *phcKLMNOP*, in the phenol- and TCE-degrading *Comamonas testosteroni* strain R5 (5). The amino acid sequences of PHE2-6 *phcKLMNOP* showed high similarities ranging from 97.9% to 100% to those of strain R5. They also had similarities ranging from 37.5% to 62.2% to those of the phenol- and TCE-degrading *Pseudomonas putida* strain CF600.

The 16S rRNA gene sequence of strain PHE2-6 has 100% identity with those of the type strains *Comamonas thiooxydans* (GenBank accession no. BBVD01000034) and *C. testosteroni* (GenBank accession no. AHIL01000001). The analysis of average nucleotide identities (ANI) based on BLAST (6) revealed that strain PHE2-6 is more closely related to *C. thiooxydans* (ANI value 97.9%, GenBank accession no. BBVD00000000) than *C. testosteroni* (ANI value 93.9%, GenBank accession no. BBJZ00000000). This result reveals that the phylogenetic affiliation of strain PHE2-6 belongs to the species *C. thiooxydans* based on the 95% threshold for species delineation (7). The strain PHE2-6 (NBRC 110656) is available from the NITE Biological Resource Center (http://www.nite.go.jp/en/nbrc/index.html).

### Nucleotide sequence accession numbers.

The draft sequence of strain PHE2-6 has been deposited in the DDBJ/EMBL/GenBank databases under the accession no. LKFB00000000. The version described in this paper is LKFB01000000.
